# Draft Genome Sequence of a Novel “*Candidatus* Liberibacter” Species Detected in a *Zanthoxylum* Species from Bhutan

**DOI:** 10.1128/MRA.00897-20

**Published:** 2020-10-01

**Authors:** Grant A. Chambers, Nerida J. Donovan, Daniel R. Bogema, Namgay Om, George A. C. Beattie, Jennifer L. Morrow, Paul Holford

**Affiliations:** a NSW Department of Primary Industries, Elizabeth Macarthur Agricultural Institute, Menangle, NSW, Australia; b Western Sydney University, School of Science, Penrith, NSW, Australia; c National Plant Protection Centre, Department of Agriculture, Ministry of Agriculture & Forests, Thimphu, Bhutan; d Western Sydney University, Hawkesbury Institute for the Environment, Penrith, NSW, Australia; Indiana University, Bloomington

## Abstract

The draft genome sequence of a novel “*Candidatus* Liberibacter” species detected in an unidentified species of *Zanthoxylum* (Rutaceae) collected in Bhutan is reported. The total length is 1,408,989 bp with 1,169 coding sequences in 96 contigs, a GC content of 37.3%, and 76 to 77% average nucleotide identity with several other “*Ca*. Liberibacter” species.

## ANNOUNCEMENT

Members of the genus *Liberibacter* are plant pathogens or nonpathogenic endophytes that are transmitted among their hosts by various psyllids. Because psyllids can harbor plant pathogens, a study of the microbiomes of psyllids associated with Rutaceae in Bhutan was performed using high-throughput sequencing (1). This revealed a novel “*Candidatus* Liberibacter” species in Cornopsylla rotundiconis and an uncharacterized species of *Cacopsylla*. Preliminary phylogenetic analysis revealed that the bacterium is closely related to “*Candidatus* Liberibacter solanacearum” and “*Candidatus* Liberibacter caribbeanus.” Here, we report the draft genome sequence of the “*Ca*. Liberibacter” isolate sampled in July 2014 from an uncharacterized *Zanthoxylum* species, a host of the psyllids, from Tsirang, Bhutan. The plant sampled exhibited blotchy mottled symptoms; however, the status of the bacterium as a pathogen or endophyte is unknown. “*Candidatus* Liberibacter africanus” was previously found in *Zanthoxylum* spp. (2).

Total DNA was extracted from petioles and midrib tissue using an Isolate II plant DNA kit (Bioline). The bacterial DNA extract was enriched using a NEBNext microbiome DNA enrichment kit (New England Biolabs) and submitted to the Ramaciotti Centre for Genomics (University of New South Wales [UNSW] Sydney, Australia) for library preparation and sequencing. A library was prepared using a Nextera DNA Flex library prep kit (Illumina), and the 2 × 150-bp paired-end library was sequenced using an Illumina NextSeq 500 platform. A total of 3.27 × 10^8^ reads with a mean length of 147 bp were generated.

**FIG 1:**
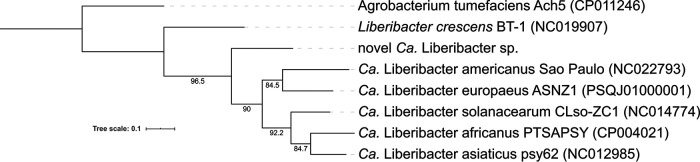
Species tree of *Liberibacter* spp. generated with 511 single-copy orthologs identified with OrthoFinder v2.4.0 (10) using default settings; individual protein sequences were aligned with MAFFT v7.471 (11) using the --auto argument. Phylogenetic trees and gene concordance factors (branch labels) were inferred with IQ-TREE v2.0.3 (12–15) with default settings. Branch support values were calculated using the ultrafast bootstrap method within IQ-TREE utilizing 1,000 replicates and showed 100% support for each branch within the tree.

The reads were analyzed with FastQC v0.11.8 and passed all relevant quality checks, were trimmed using BBDuk (3) using the parameters ktrim=r, k=23, mink=11, and hdist=1, and were assembled with SPAdes v3.13 (4) using k-mer sizes of 21, 41, 71, 101, and 127 and the --careful option. A total of 955,328 scaffolds were generated, with the largest being 122,841 bp long. Scaffolds were analyzed with BLASTn against the nucleotide database (downloaded 3 July 2020), and results were used in further analysis with BlobTools v1.1.1 (5). A BlobPlot based on the BLAST results, GC content, length, and average coverage depth was generated, and a clear cluster of scaffolds with GC content consistent with those of “*Ca*. Liberibacter” spp. (31.1 to 36.4%) and relatively high coverage depth was observed in a plot limited to the *Proteobacteria* phylum. Using this cluster as a guide, *Proteobacteria* and “no-hit” scaffolds with >20× coverage, a >200-bp length, and 35 to 50% GC content were subsequently used for individual BLASTn nucleotide searches using standard parameters and limited to the proteobacterium database. A total of 96 contigs (*N*_50_, 25,705 bp) showed similarity to *Liberibacter* species, with average GC contents of 37.3% and average coverage of ∼70.5×, forming the draft genome. The draft genome was annotated using the NCBI Prokaryotic Genome Annotation Pipeline (6, 7). The total length of the assembly was 1,408,989 bp, and the genome was predicted to have 1,169 coding sequences and 47 RNA genes. Average nucleotide identity analysis was performed using FastANI v1.0 (8) against reference sequences of “*Ca.* Liberibacter africanus” (GenBank accession number CP004021), “*Candidatus* Liberibacter americanus” (NC022793), “*Candidatus* Liberibacter asiaticus” (NC012985), and “*Ca.* Liberibacter solanacearum” (CP002371) and determined identities of 76 to 77% to each. Analysis with BUSCO v4.1.2 (9), using the proteobacteria_obd10 data set with 219 BUSCOs and default settings, showed 88% complete, 4% fragmented, 7.7% missing, and 0% duplicated BUSCOs; this low level of duplication indicates that the reads have come from a single isolate. Phylogenetic analysis (Fig. 1) shows a very high level of gene concordance and provides strong evidence that a single isolate is present. The discovery of this novel “*Ca*. Liberibacter” species is relevant to the phylogeny and origins of liberibacters and their potential impacts.

### Data availability.

The sequence data have been deposited in DDBJ/ENA/GenBank under the accession number JACETX000000000. The version described in this paper is the first version, JACETX010000000. The raw reads are deposited in the NCBI Sequence Read Archive (SRA) under the BioProject PRJNA645749.
